# Impact of diabetes mellitus in patients undergoing contemporary percutaneous coronary intervention: Results from a Korean nationwide study

**DOI:** 10.1371/journal.pone.0208746

**Published:** 2018-12-10

**Authors:** Yujin Yang, Gyung-Min Park, Seungbong Han, Yong-Giun Kim, Jon Suh, Hyun Woo Park, Ki-Bum Won, Soe Hee Ann, Shin-Jae Kim, Dae-Won Kim, Mahn-Won Park, Sung Ho Her, Sang-Gon Lee

**Affiliations:** 1 Department of Cardiology, Ulsan University Hospital, University of Ulsan College of Medicine, Ulsan, Korea; 2 Department of Applied Statistics, Gachon University, Seongnam, Korea; 3 Department of Cardiology, Soon Chun Hyang University Hospital Bucheon, Bucheon, Korea; 4 Department of Cardiology, Daejeon St. Mary's Hospital, The Catholic University of Korea, Seoul, Korea; Azienda Ospedaliero Universitaria Careggi, ITALY

## Abstract

**Objectives:**

Despite an obvious improvement in the treatment of coronary artery disease (CAD) and survival rate of patients with CAD during recent decades, diabetes mellitus (DM) is still considered a risk factor of adverse clinical outcomes in these patients. Therefore, we sought to evaluate the clinical implications of DM in patients with CAD who underwent contemporary percutaneous coronary intervention (PCI).

**Methods:**

Based on the National Health Insurance claims data in South Korea, patients aged 18 years or older who had undergone PCI for the diagnosis of CAD between 2011 and 2015 were analyzed. Patients were classified into the DM (n = 26,872) and non-DM (n = 54,243) groups. The primary endpoint was all-cause mortality, and it was compared between the two groups via a propensity score matching analysis.

**Results:**

The study population was categorized as patients with angina (n = 49,228) or acute myocardial infarction (AMI, n = 31,887). The study population had a median follow-up of 2.1 years (interquartile range, 1.1–3.2). After the propensity score matching analysis, 8,157 and 4,266 pairs of patients with angina and AMI were identified, respectively. In the matched angina group, the incidence of all-cause death was significantly higher in patients with DM (adjusted hazard ratio [aHR]: 1.30; 95% confidence interval [CI]: 1.16–1.47; p<0.001) than in those without DM. Moreover, in the matched AMI group, the incidence of all-cause death was significantly higher in patients with DM (aHR: 1.35; 95% CI: 1.19–1.53; p<0.001) than in those without DM.

**Conclusions:**

In patients undergoing contemporary PCI in Korea, the presence of DM was associated with poorer clinical outcomes.

## Introduction

The prevalence of diabetes mellitus (DM) is increasing worldwide, which is driven by both population aging and an increased prevalence of overweight and obesity [1, 2]. In particular, the prevalence of DM in Asian populations has increased rapidly in the recent decades [3]. In patients with DM, the leading cause of death is coronary artery disease (CAD) [4]. Despite an obvious improvement in the treatment of CAD and survival rate of patients with CAD [5], the mortality rate remains higher in patients with DM than in those without [6, 7]. In addition, there are several data that support that DM is an important prognostic factor of mortality and complications in patients with CAD [7, 8]. However, in contemporary real-world practice, data that can be used to assess the clinical implications of DM among patients with established CAD are limited. Therefore, using the claims data of the National Health Insurance (NHI) in South Korea, the clinical impact of DM in patients with CAD who underwent percutaneous coronary intervention (PCI) in Korea was evaluated.

## Methods

### Data sources

As described in detail previously [9], in South Korea, all healthcare providers had to join the NHI system on a fee-for-service basis. The Health Insurance Review & Assessment Service (HIRA) is a quasi-governmental organization that systematically reviews medical fees to minimize the risk of redundant and unnecessary medical services. Accordingly, all NHI claims records are reviewed by the HIRA. The current study analyzed data from the January 2011 to June 2016 claims records of the HIRA. The diagnosis codes of the International Classification of Diseases, 10th Revision (ICD-10) were used. In addition, specific information about drugs, devices, and procedures was obtained using codes from the HIRA database [9]. Since the claims data of the HIRA were fully anonymized, this study was approved by the local institutional review board of Ulsan University Hospital, Ulsan, Korea, which waived the requirement for informed consent.

### Study population

Based on the claims database of the HIRA from July 2011 to June 2015, we identified patients aged 18 years and older who had undergone PCI (M6551, M6552, M6561–4, M6571, and M6572) for the diagnosis of CAD (ICD-10 codes I20.X–I25.X). To ensure patients’ first episode of CAD, patients with at least 6 months of eligibility prior to the index day were selected. Patients were also excluded if the HIRA database indicated that patients had a previous history of CAD (ICD-10 codes I20.X–25.X) within 6 months of the index day. Furthermore, patients were categorized as either those with acute myocardial infarction (AMI) and angina pectoris, and separate analyses were conducted for each group. AMI was defined using the ICD-10 codes (I21.X–I22.X) in the hospital discharge databases of the HIRA [9].

### Study variables

DM was defined as patients who were assigned with the ICD-10 codes for those with DM (E10.0, E10.1, E10.6, E10.8-E11.1, E11.6, E11.8-E12.1, E12.6, E12.8-E13.1, E13.6, E13.8-E14.1, E14.6, E14.8, and E14.9); those with DM and chronic complications (E10.2-E10.5, E10.7, E11.2-E11.5, E11.7, E12.2-E12.5, E12.7, E13.2-E13.5, E13.7, E14.2-E14.5, and E14.7); or those who use anti-diabetic medications from the medication codes in the HIRA database within 6 months of the index day [9, 10].

Within 6 months of the index day, the ICD-10 codes were used to identify other comorbidities, such as hyperlipidemia, hypertension, congestive heart failure, arrhythmia, valvular disease, peripheral vascular disease, cerebrovascular disease, chronic pulmonary disease, moderate to severe liver disease, renal disease, cancer, and rheumatic disease [9–11]. The Charlson comorbidity index was obtained using the ICD-10 codes [9–11]. In the HIRA database, all prescribed medications were recorded with rigorous accuracy. Moreover, patients who use anti-hypertensive and anti-hyperlipidemic drugs were considered to have hypertension and hyperlipidemia, respectively. Furthermore, we identified the use of medications, such as antiplatelet agents, statins, beta-blockers, and angiotensin-converting enzyme inhibitors (ACEIs) or angiotensin receptor blockers (ARBs) [9].

We assigned claims as drug-eluting stents (DES) if DES device codes (J5083XXX) were used. The claims were designated as bare metal stents (BMS) if there were BMS device codes (J5231XXX). The claims were also classified as a non-stent coronary balloon angioplasty if device codes did not include any code indicating a DES or BMS [9].

### Clinical outcomes

All-cause deaths were identified by all in- and out-patient claims that indicated death. Coronary revascularizations were identified using the procedure codes of PCI (M6551, M6552, M6561-4, M6571, and M6572) and coronary artery bypass surgery (O1641, O1642, O1647, OA641, OA642, and OA647) from the HIRA database. In the current study, for the evaluation of clinical outcomes, the HIRA database was used until June 2016. In patients with multiple events, the first event was considered to be the component of the composite outcome [9, 11].

### Statistical analysis

We conducted separate analyses of the angina and AMI groups. Baseline patient characteristics and comorbidities were presented as mean ± standard deviation or frequency (%) for continuous or categorical variables, respectively. Continuous variables with normality were compared using the student’s t-test, and those without normality were compared using the Mann–Whitney U test. Categorical data were compared using the chi-square or Fisher’s exact test. Cumulative incidence rates for all-cause death between the DM and non-DM groups were estimated using the Kaplan–Meier method. Furthermore, we compared the cumulative incidence rates between the DM and non-DM groups using the log-rank test. The generalized estimating equations and the Cox proportional hazards regression model were used to identify adjusted DM effects for the in-hospital mortality or the time to event data, respectively. In particular, to reduce the impact of potential confounding effects between the comparison groups, significant differences in the baseline characteristics were adjusted using the propensity score matching method. The propensity scores were obtained nonparametrically using age, gender, comorbidities, type and number of stents, and the Charlson comorbidity index. Nonparametric propensity score estimation was useful because there was no need to fit the fully corrected parametric model. Propensity score matching was performed with the nearest-neighbor matching using a caliper size of 0.2 multiplied by the standard deviation for linearly transformed propensity scores (logit transformation). The balance of covariates in the matched groups was evaluated by measuring their standardized differences in means. All standardized differences in the baseline variables were less than 0.05 (5%). Thus, we examined whether all pretreatment variables were balanced between the two comparison groups. In addition, we conducted the paired t-test or the McNemar test for continuous or categorical variables, respectively, to assess the covariate balance between the two matched groups. In the propensity score-matched cohort, the in-hospital mortality rates were compared using the generalized estimating equations. For the time to event data, the Cox regression model with robust standard errors was used to accommodate the clustering of matched pairs. The R packages of “MatchIt,” “geepack,” and “survival” were used for the propensity score matching, generalized estimating equation fitting, and Cox model fitting, respectively. All data analyses were performed using the R software version 3.3.1 (R Foundation for Statistical Computing, Vienna, Austria; www.r-project.org). A p value <0.05 was considered significant for all two-sided tests.

## Results

### Study population and characteristics

**Fig 1:**
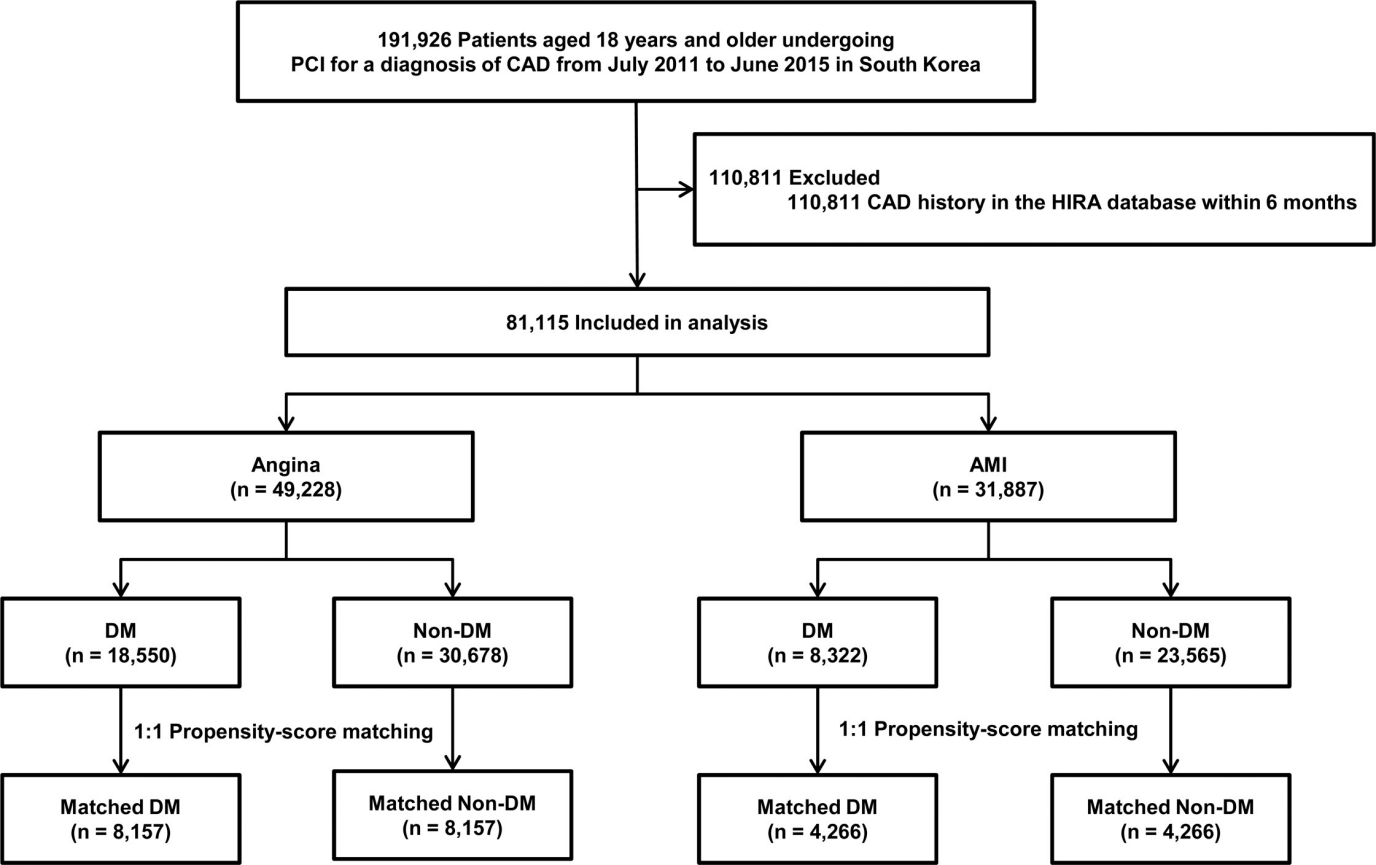
Overview of the study population. CAD = coronary artery disease; DM = diabetes mellitus; HIRA = Health Insurance Review & Assessment Service; AMI = acute myocardial infarction; PCI = percutaneous coronary intervention.

Between July 2011 and June 2015, a total of 191,926 patients aged 18 years and older who were diagnosed with CAD and underwent PCI were identified from the claims database of HIRA. Among them, 81,115 met the eligibility criteria, and they were then included in the analysis. Table 1 shows the baseline characteristics of the study participants. The mean age of the study participants was 64.4 ± 12.2 years and 56,576 (69.7%) were men. DM, hyperlipidemia, and hypertension were observed in 26,872 (33.1%), 30,675 (37.8%), and 45,389 (56.0%) patients, respectively. In PCI procedures, DESs were the most frequently used devices (n = 75,600, 93.2%), followed by balloon angioplasty (n = 4,479, 5.5%) and BMSs (n = 1,036, 1.3%). At discharge, antiplatelet agents, statins, beta-blockers, and ACEIs or ARBs were provided to 80,575 (99.3%), 71,411 (88.0%), 57,429 (70.8%), and 54,418 (67.1%) patients, respectively. According to the presence of DM, patients were classified into the DM and non-DM groups (Fig 1). In addition, specific anti-diabetic agents were presented in S1 Table. The number of diabetic patients with insulin treatment was 5,813 patients. In the overall population, during the follow-up period (median, 2.1 years; interquartile range, 1.1–3.2), 13,340 patients had 5,849 deaths and 7,881 coronary revascularizations (S2 Table).

**Table 1:** Characteristics of patients undergoing percutaneous coronary intervention according to the presence of diabetes mellitus.

	Angina (n = 49,228)			AMI (n = 31,887)		
Characteristics	DM (n = 18,550)	Non-DM (n = 30,678)	p value	DM (n = 8,322)	Non-DM (n = 23,565)	p value
Age, years	66.7 ± 10.4	64.2 ± 12.0	<0.001	66.1 ± 11.5	62.1 ± 13.4	<0.001
Gender (male), no. (%)	11,425 (61.6%)	21,343 (69.6%)	<0.001	5,572 (67.0%)	18,236 (77.4%)	<0.001
Number of participants, no. (%)				0.812			0.510
July 2011 to June 2012	4,148 (22.4%)	6,957 (22.7%)		2,152 (25.9%)	5,916 (25.1%)	
July 2012 to June 2013	4,479 (24.1%)	7,404 (24.1%)		2,098 (25.2%)	5,922 (25.1%)	
July 2013 to June 2014	4,889 (26.4%)	8,090 (26.4%)		2,062 (24.8%)	5,914 (25.1%)	
July 2014 to June 2015	5,034 (27.1%)	8,227 (26.8%)		2,010 (24.2%)	5,813 (24.7%)	
Comorbidities, no. (%)							
Hyperlipidemia	12,559 (67.7%)	10,037 (32.7%)	<0.001	4,419 (53.1%)	3,660 (15.5%)	<0.001
Hypertension	15,237 (82.1%)	16,014 (52.2%)	<0.001	6,140 (73.8%)	7,998 (33.9%)	<0.001
Congestive heart failure	1,916 (10.3%)	1,626 (5.3%)	<0.001	500 (6.0%)	483 (2.0%)	<0.001
Arrhythmia	1,905 (10.3%)	2,243 (7.3%)	<0.001	417 (5.0%)	581 (2.5%)	<0.001
Valvular disease	107 (0.6%)	144 (0.5%)	0.117	19 (0.2%)	37 (0.2%)	0.222
Peripheral vascular disease	3,136 (16.9%)	2,648 (8.6%)	<0.001	1,238 (14.9%)	1,276 (5.4%)	<0.001
Cerebrovascular disease	3,764 (20.3%)	3,279 (10.7%)	<0.001	1,144 (13.7%)	1,324 (5.6%)	<0.001
Chronic pulmonary disease	3,630 (19.6%)	4,624 (15.1%)	<0.001	1,422 (17.1%)	2,600 (11.0%)	<0.001
Moderate to severe liver disease	16 (0.1%)	10 (0.03%)	0.015	11 (0.1%)	4 (0.02%)	<0.001
Renal disease	1,941 (10.5%)	734 (2.4%)	<0.001	575 (6.9%)	230 (1.0%)	<0.001
Cancer	769 (4.1%)	662 (2.2%)	<0.001	292 (3.5%)	373 (1.6%)	<0.001
Rheumatic disease	52 (0.3%)	48 (0.2%)	0.004	16 (0.2%)	34 (0.1%)	0.336
Charlson comorbidity index	2.48 ± 1.34	0.78 ± 1.03	<0.001	2.21 ± 1.26	0.51 ± 0.84	<0.001
Type of treatment, no. (%)			0.021			0.033
Drug-eluting stents	17,212 (92.8%)	28,655 (93.4%)		7,718 (92.7%)	22,015 (93.4%)	
Bare metal stents	230 (1.2%)	324 (1.1%)		122 (1.5%)	360 (1.5%)	
Balloon angioplasty (no stent)	1,108 (6.0%)	1,699 (5.5%)		482 (5.8%)	1,190 (5.0%)	
Number of stents per person	1.46 ± 0.69	1.39 ± 0.65	<0.001	1.42 ± 0.64	1.34 ± 0.59	<0.001
Medications at discharge, no. (%)						
Antiplatelet agents	18,348 (98.9%)	30,473 (99.3%)	<0.001	8,277 (99.5%)	23,477 (99.6%)	0.048
Statins	15,561 (83.9%)	27,049 (88.2%)	<0.001	7,352 (88.3%)	21,449 (91.0%)	<0.001
Beta-blockers	11,867 (64.0%)	19,602 (63.9%)	0.869	6,635 (79.7%)	19,325 (82.0%)	<0.001
ACEI/ARB	12,102 (65.2%)	18,633 (60.7%)	<0.001	6,157 (74.0%)	17,526 (74.4%)	0.493

Data were expressed as n (%) and mean ± SD.

ACEI = angiotensin-converting enzyme inhibitor; AMI = acute myocardial infarction; ARB = angiotensin receptor blocker; DM = diabetes mellitus

### DM versus non-DM in angina

**Fig 2:**
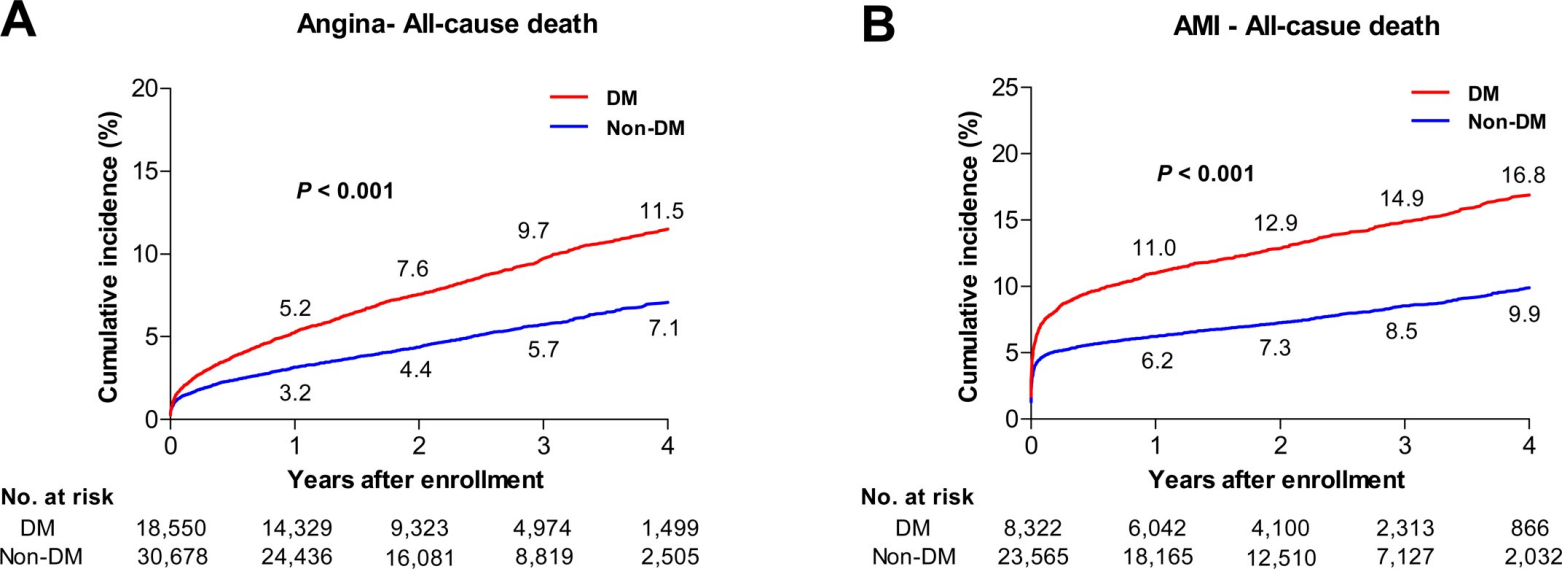
Cumulative incidence rates for all-cause deaths in the study population. Cumulative incidence rates for (A) all-cause death in patients with angina and (B) in those with acute myocardial infarction. The numbers in each figure represent the cumulative incidence rates at each time point. AMI = acute myocardial infarction.

According to diagnosis, the study participants were classified as patients with angina pectoris (n = 49,228). Among them, patients were categorized into the DM (n = 18,550) and non-DM (n = 30,678) groups. Patients with DM were older and had more comorbidities than those without DM (Table 1). Fig 2 shows the cumulative incidence rates for all-cause deaths between the two groups. After the propensity score matching, there were 8,157 matched pairs. In the matched cohort, no significant differences in terms of covariates were observed between the two groups (Table 2). During the follow-up period (median, 2.1 years; interquartile range, 1.1–3.1), 2,435 patients had 1,088 deaths and 1,414 coronary revascularizations (S3 Table). There was no significant difference in terms of the incidence of in-hospital mortality between the two groups (adjusted odds ratio [aOR] of DM: 1.31; 95% confidence interval [CI]: 0.99–1.74; p = 0.062). However, the occurrence of all-cause death (adjusted hazard ratio [aHR] of DM: 1.30; 95% CI: 1.16–1.47; p<0.001) and the composite of death and recurrent coronary revascularization (aHR: 1.20; 95% CI: 1.11–1.30; p<0.001) was significantly higher in the DM group than in the non-DM group (Table 3). In DM group, matched diabetic patients with insulin treatment had poorer short- and long-term clinical outcomes compared with those without (Table 4).

**Table 2:** Characteristics of the propensity score-matched patients according to the presence of diabetes mellitus.

	Angina (n = 8,157 pairs)					AMI (n = 4,266 pairs)		
Characteristics	DM (n = 8,157)	Non-DM (n = 8,157)			p value	DM (n = 4,266)	Non-DM (n = 4,266)	p value
Age, years	66.6 ± 10.5	66.8 ± 11.4			0.860	65.8 ± 11.7	66.1 ± 12.5	0.360
Gender (male), no. (%)	5,080 (62.3%)	5,109 (62.6%)			0.327	2,902 (68.0%)	2,895 (67.9%)	0.570
Comorbidities, no. (%)								
Hyperlipidemia	4,702 (57.6%)	4,410 (54.1%)			0.426	1,857 (43.5%)	1,661 (38.9%)	0.896
Hypertension	6,142 (75.3%)	5,852 (71.7%)			0.131	2,749 (64.4%)	2,609 (61.2%)	0.244
Congestive heart failure	745 (9.1%)	694 (8.5%)			0.164	214 (5.0%)	197 (4.6%)	0.155
Arrhythmia	839 (10.3%)	844 (10.3%)			0.759	197 (4.6%)	199 (4.7%)	0.718
Valvular disease	47 (0.6%)	47 (0.6%)			0.606	11 (0.3%)	13 (0.3%)	0.540
Peripheral vascular disease	1,197 (14.7%)	1,130 (13.9%)			0.297	546 (12.8%)	484 (11.3%)	0.712
Cerebrovascular disease	1,484 (18.2%)	1,428 (17.5%)			0.788	500 (11.7%)	509 (11.9%)	0.094
Chronic pulmonary disease	1,647 (20.2%)	1,680 (20.6%)			0.032	697 (16.3%)	747 (17.5%)	0.927
Moderate to severe liver disease	7 (0.1%)	6 (0.1%)			0.096	4 (0.1%)	4 (0.1%)	0.724
Renal disease	500 (6.1%)	339 (4.2%)			0.183	165 (3.9%)	108 (2.5%)	0.478
Cancer	302 (3.7%)	302 (3.7%)			0.478	126 (3.0%)	108 (2.5%)	0.460
Rheumatic disease	21 (0.3%)	22 (0.3%)			0.760	9 (0.2%)	14 (0.3%)	0.677
Charlson comorbidity index	1.96 ± 1.14	1.87 ± 1.02			0.256	1.73 ± 1.01	1.68 ± 0.92	0.663
Type of treatment, no. (%)								
Drug-eluting stents	7,583 (93.0%)	7,614 (93.3%)			0.711	3,959 (92.8%)	3,962 (92.9%)	0.502
Bare metal stents	104 (1.3%)	99 (1.2%)			0.160	65 (1.5%)	67 (1.6%)	0.027
Number of drug-eluting stents	1.35 ± 0.74	1.33 ± 0.71			0.156	1.33 ± 0.70	1.31 ± 0.67	0.549

Data were expressed as n (%) and mean ± SD.

ACEI = angiotensin-converting enzyme inhibitor; AMI = acute myocardial infarction; ARB = angiotensin receptor blocker; DM = diabetes mellitus

**Table 3:** Clinical outcomes in patients who underwent percutaneous coronary intervention according to the presence of diabetes mellitus.

Propensity score matching analysis	Angina (n = 8,157 pairs)		AMI (n = 4,266 pairs)	
	DM compared with non-DM		DM compared with non-DM	
	Odds ratio (95% CI)	p value	Odds ratio (95% CI)	p value
In-hospital mortality	1.31 (0.99–1.74)	0.062	1.41 (1.16–1.70)	<0.001
	Hazard ratio (95% CI)	p value	Hazard ratio (95% CI)	p value
All-cause death	1.30 (1.16–1.47)	<0.001	1.35 (1.19–1.53)	<0.001
Death/coronary revascularization	1.20 (1.11–1.30)	<0.001	1.32 (1.21–1.45)	<0.001

AMI = acute myocardial infarction; CI = confidence interval; DM = diabetes mellitus

**Table 4:** Clinical outcomes in patients with diabetes mellitus who underwent percutaneous coronary intervention according to the treatment of insulin.

Propensity score matching analysis	Angina (n = 3,948 pairs)		AMI (n = 1,452 pairs)	
	DM with insulin treatment compared with DM without		DM with insulin treatment compared with DM without	
	Odds ratio (95% CI)	p value	Odds ratio (95% CI)	p value
In-hospital mortality	1.55 (1.11–2.16)	0.010	1.48 (1.11–1.97)	0.008
	Hazard ratio (95% CI)	p value	Hazard ratio (95% CI)	p value
All-cause death	1.54 (1.34–1.78)	<0.001	1.61 (1.36–1.91)	<0.001
Death/coronary revascularization	1.33 (1.20–1.47)	<0.001	1.39 (1.22–1.58)	<0.001

AMI = acute myocardial infarction; CI = confidence interval; DM = diabetes mellitus

### DM versus non-DM in AMI

We also analyzed the clinical outcomes in patients with AMI (n = 31,887). Patients were classified into the DM (n = 8,322) and non-DM (n = 23,565) groups. Patients with DM were older and had more comorbidities than those without DM (Table 1). The cumulative incidence rates for all-cause deaths between the two groups are presented in Fig 2. Among the 4,266 propensity score-matched pairs, no significant differences were observed in terms of covariates between both groups (Table 2). During the follow-up period (median, 2.0 years; interquartile range, 1.0–3.2), 1,863 patients had 966 deaths and 967 coronary revascularizations (S3 Table). The incidence of all-cause death was significantly higher in patients with DM (aHR: 1.35; 95% CI: 1.19–1.53; p<0.001) than in those without. In addition, the occurrence of in-hospital mortality (aHR: 1.41; 95% CI: 1.16–1.70; p<0.001) and composite of death and recurrent coronary revascularization (aHR: 1.32; 95% CI: 1.21–1.45; p<0.001) was higher in patients with DM than in those without (Table 3). In DM group, the poorer short- and long-term clinical outcomes were observed in matched diabetic patients with insulin treatment compared with those without (Table 4).

## Discussion

In the present analysis that used data from the NHI claims database in South Korea, the presence of DM was associated with poorer clinical outcomes in patients who underwent PCI for established CAD regardless of clinical presentations.

In the recent decades, there have been remarkable advancements in adjuvant pharmacologic agents and devices for treating CAD. Optimal medical therapy has been suggested as an initial treatment strategy and recommended for all patients with CAD [12, 13]. Particularly, in patients with stable CAD, optimal medical therapy had comparable clinical benefits with coronary revascularization [14, 15]. Furthermore, in those with acute coronary syndrome, new P2Y12 agents significantly improved clinical outcomes [16, 17]. On the other hand, there has also been a significant improvement in stent design and the development of new drugs and the drug-carrier systems of devices. Based on these enhanced properties, contemporary DESs had better clinical efficacy and safety than BMSs and early-generation DESs [18]. Previous studies have shown that DM had a greater adverse clinical impact on short- and long-term clinical outcomes [6, 7, 19–23]. However, considering the contemporary real-world practice, there are limited data to evaluate the clinical implications of DM. Therefore, the present study was designed. Well-controlled and reliable data from the HIRA database in Korea (i.e., a quasigovernmental organization) enabled qualified analyses for the clinical impact of DM in patients undergoing PCI for the established CAD [9, 11, 24].

For patients with angina, coronary angioplasty in diabetic patients showed a similar procedural success without increased in-hospital mortality compared with non-diabetic patients. However, DM was associated with a worse long-term prognosis [19]. Even in the PCI era, the procedural success rates in patients with DM and those without DM are comparable. However, patients with DM had a higher incidence of long-term adverse clinical outcomes [20]. Consistent with these studies, the present study showed that in-hospital mortality in patients with angina was comparable between the DM and non-DM groups. However, long-term clinical outcomes were poorer in DM group. On the contrary, in earlier studies on AMI, patients with DM showed not only increased in-hospital mortality but also worse long-term prognosis [21, 22]. Despite an improvement in the treatment of AMI, in-hospital and long-term mortality rates were still higher in patients with DM [7, 23]. Moreover, the present study, which reflects contemporary practice, showed that patients with concurrent DM and AMI had increased in-hospital and long-term mortalities. Therefore, in patients with both DM and angina, we focused on post-PCI management. In patients with AMI, preemptive prevention, early detection, and proper treatment for DM are required to improve prognosis.

DM adversely affects the outcome and course of CAD. In patients with DM, platelet hyperactivity, reduced fibrinolytic capacity, increased concentrations of hemostatic proteins, and endothelial dysfunction promote atherosclerosis and increase the risk of thrombotic vascular events [25–27]. However, an even more important thing is that the adherence to guidelines could improve clinical outcomes in patients with DM. A previous long-term study has shown that a targeted, intensified, multifactorial intervention reduces the risk of cardiovascular and microvascular events in patients with DM [28]. A randomized trial has also observed the resolution of myocardial ischemia that resulted from a more intensive treatment of cardiovascular risk factors [29]. Therefore, to prevent future CAD in this high-risk population, aggressive preventive strategies according to current guidelines must be implemented.

The anatomical SYNTAX (SYNergy between percutaneous coronary intervention with TAXus and cardiac surgery) score was developed to help physicians decide the optimal revascularization modality in patients with complex CAD [30]. However, this lesion-based scoring system showed to have a lower ability to predict mortality compared with scoring systems using clinical variables. To overcome these limitations, the clinical SYNTAX score combining the SYNTAX score with a simple clinical risk score incorporating age, ejection fraction, and creatinine clearance was advocated [31]. However, in a validation study with a patient-level pooled analysis of 6,081 patients treated with DES (75% newer-generation DES), both SYNTAX score and DM were associated with 2-year major adverse cardiac events defined as the composite of cardiac death, myocardial infarction, and clinically indicated target lesion revascularization (p <0.001 and p = 0.028, respectively). In addition, compared with patients without DM, those with DM had higher risks of 2-year major adverse cardiac events (aHR: 1.25; 95% CI: 1.03–1.53; p = 0.026) without significant interaction with SYNTAX score [32]. Therefore, these findings imply that DM is still an important risk factor in patients with complex CAD.

A previous large randomized trial compared CABG and PCI with DES in 1,900 patients with DM and multi-vessel disease. CABG showed a significantly lower 5-year event rates for the composite of all-cause death, MI, and stroke compared to PCI with DES (18.7% in the CABG group versus 26.6% in the PCI group, p = 0.005) [33]. In a prespecified subgroup analysis for 452 patients with DM of the SYNTAX trial, the 5-year event rates of the composite of death, stroke, MI and repeat revascularization were also significantly lower in the CABG group compared with PCI group (29.0% versus 46.5%, p<0.001) [34]. Consequently, the current guideline recommends CABG as the revascularization modality of choice in patients with DM and multi-vessel or left main disease [35]. However, evidence supporting the guideline was largely based on the early-generation DES. A recent network meta-analysis suggested that PCI with everolimus-eluting stent was associated with similar risk of long-term death compared with CABG [36]. Therefore, future randomized clinical trials are required to compare CABG and PCI with newer-generation DES in patients with DM.

The present study had several limitations. First, our study was based on administrative data from the HIRA database in South Korea. The present study lacked clinical data and medical examination results, which is similar to previous studies that used data from administrative databases. Thus, our findings might be limited by uncertainties in terms of unmeasured confounding variables that may affect the management of patients [9, 11]. Second, although we used a database from a quasi-governmental organization, there is a possibility that these data did not fully reflect patient outcomes. In addition, we did not specify the cause of death. Finally, the present study only included a population in Korea. Ethnic differences in CAD and clinical differences in patients with DM have been observed between the Asian and Western populations [37]. Therefore, this may limit the generalizability of our findings to other ethnic groups.

## Conclusions

Despite a significant progress in the treatment of patients with CAD, DM is still an independent risk factor for adverse clinical outcomes in patients who underwent contemporary PCI in Korea. Our findings highlight the importance of appropriate and aggressive strategies that reduce the risk of CAD in patients with DM.

## Supporting information

S1 Table Frequency of anti-diabetic agents. (DOCX)

S2 Table Clinical outcomes according to the presence of diabetes mellitus in overall population. (DOCX)

S3 Table Clinical outcomes according to the presence of diabetes mellitus in matched population. (DOCX)

## Abbreviations

ACEI: angiotensin-converting enzyme inhibitor
AMI: acute myocardial infarction
ARB: angiotensin receptor blocker
BMS: bare metal stent
CAD: coronary artery disease
CI: confidence interval
DES: drug-eluting stent
DM: diabetes mellitus
NHI: National Health Insurance
HIRA: Health Insurance Review & Assessment Service
HR: hazard ratio
ICD-10: International Classification of Diseases, 10th Revision
OR: odds ratio
PCI: percutaneous coronary intervention
