# Supplementary material for: Impact of diabetes mellitus in patients undergoing contemporary percutaneous coronary intervention: Results from a Korean nationwide study
Source: PLoS One. 2018 Dec 10;13(12):e0208746. doi: 10.1371/journal.pone.0208746 (PMC6287858; doi:10.1371/journal.pone.0208746)
Supplement: S1 Table — (DOCX) [file pone.0208746.s001.docx]

**S1 Table. Frequency of anti-diabetic agents.**

| Frequency of anti-diabetic medications | Overall (n=26,872) | | |
| --- | --- | --- | --- |
|  | DM in Angina  (n=18,550) | DM in AMI  (n=8,322) | P Value |
| Insulin | 4,284 (23.1%) | 1,529 (18.4%) | <0.001 |
| Sulfonylurea | 8,845 (47.7%) | 4,483 (53.9%) | <0.001 |
| Metformin | 11,043 (59.5%) | 5,323 (64.0%) | <0.001 |
| α-Glucosidase inhibitor | 1,805 (9.7%) | 932 (11.2%) | <0.001 |
| Thiazolidinedione | 861 (4.6%) | 360 (4.3%) | 0.254 |
| Glinide | 545 (2.9%) | 194 (2.3%) | 0.005 |
| Dipeptidyl peptidase-4 inhibitor | 5,040 (27.2%) | 2,218 (26.7%) | 0.381 |
| Sodium-glucose co-transporter-2 inhibitor | 14 (0.1%) | 6 (0.1%) | 0.999 |
| Glucagon-like peptide-1 receptor agonist | 7 (0.04%) | 0 (0%) | 0.108 |

Values are presented as n (%).

AMI = acute myocardial infarction; DM = diabetes mellitus
