# Supplementary material for: Impact of diabetes mellitus in patients undergoing contemporary percutaneous coronary intervention: Results from a Korean nationwide study
Source: PLoS One. 2018 Dec 10;13(12):e0208746. doi: 10.1371/journal.pone.0208746 (PMC6287858; doi:10.1371/journal.pone.0208746)
Supplement: S2 Table — (DOCX) [file pone.0208746.s002.docx]

**S2 Table. Clinical outcomes according to the presence of diabetes mellitus in overall population.**

| Clinical outcomes during the follow-up period  (median, 2.1 years; interquartile range, 1.1–3.2) | Angina (n=49,228) | | |
| --- | --- | --- | --- |
|  | DM  (n=18,550) | Non-DM  (n=30,678) | P Value |
| In-hospital mortality | 282 (1.5%) | 329 (1.1%) | <0.001* |
| All-cause death | 1,470 (7.9%) | 1,457 (4.7%) | <0.001† |
| Coronary revascularization | 1,768 (9.5%) | 2,594 (8.5%) | <0.001† |
| Death/coronary revascularization | 3,136 (16.9%) | 3,959 (12.9%) | <0.001† |
| Clinical outcomes during the follow-up period  (median, 2.1 years; interquartile range, 1.0–3.2) | AMI (n=31,887) | | |
|  | DM  (n=8,322) | Non-DM  (n=23,565) | P Value |
| In-hospital mortality | 516 (6.2%) | 967 (4.1%) | <0.001* |
| All-cause death | 1,110 (13.3%) | 1,812 (7.7%) | <0.001† |
| Coronary revascularization | 1,067 (12.8%) | 2,452 (10.4%) | <0.001† |
| Death/coronary revascularization | 2,080 (25.0%) | 4,165 (17.7%) | <0.001† |

Values are presented as n (%). P-values were calculated using the logistic regression* and log-rank test†.

AMI = acute myocardial infarction; DM = diabetes mellitus
