# Supplementary material for: Impact of diabetes mellitus in patients undergoing contemporary percutaneous coronary intervention: Results from a Korean nationwide study
Source: PLoS One. 2018 Dec 10;13(12):e0208746. doi: 10.1371/journal.pone.0208746 (PMC6287858; doi:10.1371/journal.pone.0208746)
Supplement: S3 Table — (DOCX) [file pone.0208746.s003.docx]

**S3 Table. Clinical outcomes according to the presence of diabetes mellitus in matched population.**

| Clinical outcomes during the follow-up period  (median, 2.1 years; interquartile range, 1.1–3.1) | Angina (n=8,157 pairs) | | |
| --- | --- | --- | --- |
|  | DM  (n=8,157) | Non-DM  (n=8,157) | P Value |
| In-hospital mortality | 111 (1.4%) | 85 (1.0%) | 0.062* |
| All-cause death | 611 (7.5%) | 477 (5.8%) | <0.001† |
| Coronary revascularization | 734 (9.0%) | 680 (8.3%) | 0.051† |
| Death/coronary revascularization | 1,312 (16.1%) | 1,123 (13.8%) | <0.001† |
| Clinical outcomes during the follow-up period  (median, 2.0 years; interquartile range, 1.0–3.2) | AMI (n=4,266 pairs) | | |
|  | DM  (n=4,266) | Non-DM  (n=4,266) | P Value |
| In-hospital mortality | 269 (6.3%) | 195 (4.6%) | <0.001* |
| All-cause death | 550 (12.9%) | 416 (9.8%) | <0.001† |
| Coronary revascularization | 543 (12.7%) | 424 (9.9%) | <0.001† |
| Death/coronary revascularization | 1,045 (24.5%) | 818 (19.2%) | <0.001† |

Values are presented as n (%).P-values were calculated using the generalized estimating equations* and Cox regression with robust variance estimator†.

AMI = acute myocardial infarction; DM = diabetes mellitus
